# Research on the Flow Characteristics and Reaction Mechanisms of Lateral Flow Immunoassay under Non-Uniform Flow

**DOI:** 10.3390/s24061989

**Published:** 2024-03-20

**Authors:** Xuyan Zhao, Yuan Zhang, Qunfeng Niu, Li Wang, Chenglong Xing, Qiao Wang, Hui Bao

**Affiliations:** 1 College of Information Science and Engineering, Henan University of Technology, Zhengzhou 450001, China; zhaoxuyan@stu.haut.edu.cn (X.Z.);; 2 College of Electrical Engineering, Henan University of Technology, Zhengzhou 450001, China; hautwangli@haut.edu.cn (L.W.);

**Keywords:** lateral flow immunoassay (LFIA), non-uniform flow, sandwich LFIA, competitive LFIA, finite element method

## Abstract

Lateral flow immunoassay (LFIA) is extensively utilized for point-of-care testing due to its ease of operation, cost-effectiveness, and swift results. This study investigates the flow dynamics and reaction mechanisms in LFIA by developing a three-dimensional model using the Richards equation and porous media transport, and employing numerical simulations through the finite element method. The study delves into the transport and diffusion behaviors of each reaction component in both sandwich LFIA and competitive LFIA under non-uniform flow conditions. Additionally, the impact of various parameters (such as reporter particle concentration, initial capture probe concentrations for the T-line and C-line, and reaction rate constants) on LFIA performance is analyzed. The findings reveal that, in sandwich LFIA, optimizing parameters like increasing reporter particle concentration and initial capture probe concentration for the T-line, as well as adjusting reaction rate constants, can effectively enhance detection sensitivity and broaden the working range. Conversely, in competitive LFIA, the effects are inverse. This model offers valuable insights for the design and enhancement of LFIA assays.

## 1. Introduction

Lateral flow immunoassay (LFIA) has been widely used in recent years as a mainstream instant diagnostic method due to its advantages of easy operation, low material cost, and fast detection speed [1,2]. It has been widely applied in medical diagnosis, food safety, environmental monitoring, and other fields [3,4,5,6,7,8]. Large molecular analytes such as proteins are typically tested using sandwich assays, while small molecule analytes such as drugs of abuse are analyzed using competitive or inhibitory assays [9,10,11]. There may be multiple forms of reaction patterns, but they all have a common feature: reporter particles are captured by reagents immobilized on the test and control lines to form complexes, which generate visual signals within minutes. LFIA can easily obtain qualitative or semi-quantitative results, but compared to large quantitative detection instruments, it has many limitations in sensitivity.

Researchers have made many innovative efforts to improve the sensitivity of LFIA, such as inventing new reporter particles [12,13], optimizing membrane properties [14,15], improving experimental reagents [16,17], modifying LFIA geometry [18,19,20], and using different technologies [21,22,23]. These innovations have indeed enhanced the detection sensitivity. However, the development of lateral flow immunoassay test strips from the design stage to product development and final manufacturing is a process that applies principles of biology, chemistry, physics, and engineering. Structural and material changes require a considerable amount of experimentation [24].

In this context, computer simulation has become a practical tool to reduce the large amount of experimental work required for LFIA development. Simulation models of LFIA will allow developers to design and explore different architectures, materials, and analysis formats to seek better binding efficiency and improved detection sensitivity. Furthermore, running these simulation experiments significantly reduces development costs and time. Gasperino et al.’s review summarizes existing models [25], emphasizing the importance of computational models in quantifying performance and predicting future trends. Qian pioneered the establishment of mathematical models for sandwich and competitive LFIA [26,27], including a complete set of coupled reactions considering uniform and constant fluid velocity through the test lines. Berli proposed a simple mathematical model that condenses the main system parameters into two dimensionless parameters [28], relative fluid velocity, and relative analyte concentration, to quantitatively describe the dynamics of analyte capture in LFIA. Sotnikov used analytical (non-numerical) methods to calculate the dynamics of immune complex formation in continuous flow systems and also proposed an analysis of competitive LFIA under non-equilibrium conditions [29], obtaining symbolic solutions of differential equations describing the system [30]. Liu et al. established a model where reporter particles can bind to multiple target molecules and also developed a method to convert LFIA design parameters between simulation and experimentation [31,32]. Furthermore, they combined Langmuir surface reaction with convection–diffusion reaction equations to establish a thickness model for LFIA [33]. Asadi conducted a numerical study of spontaneous self-suction of shearing-related fluids using an improved Richards equation, showing that the average velocity of LFIA on the test line is strongly influenced by the microstructure of the absorption pad [34]. Schaumburg modeled LFIA using the finite element method in a Python environment and discussed its application in different dimensions [20].

Inspired by the work of Schaumburg and Liu, expanded on Qian’s numerical model and the COMSOL model established by Ed Fontes [35], we develop a 3D model of LFIA within the framework of porous media transport phenomena in COMSOL. Finite element simulations of sandwich LFIA and competitive LFIA were conducted to analyze a complete set of biochemical reactions involved in LFIA throughout the entire 3D domain. This included capillary-driven flow velocities, transport and diffusion of each reaction component under non-uniform flow, and the effects of different parameters (analyte concentration, reporter particle concentration, initial capture probe concentrations for T-line and C-line, reaction rate constants) on LFIA performance.

## 2. Mathematical Modeling

In this section, we use equations to simulate all the transport phenomena related to the performance of LFIA, including fluid dynamics, solute transport, and reaction kinetics. The model is based on the classical approach of porous media, where the microstructural properties of the matrix are represented by macroscopic parameters such as porosity, pore size, and permeability.

### 2.1. Fluid Dynamics Model

The lateral flow immunoassay strip is a porous structure, and the interaction between the liquid and the pore walls causes capillary forces that drive the liquid forward. In this paper, the Richards equation is used to describe the fluid dynamics model of capillary seepage in porous media. The Richards equation is primarily used to analyze flow in variably saturated porous media. In this type of flow, as the fluid passes through the porous medium, it fills some pores and drains from others, leading to changes in hydraulic properties. The specific expression is as follows:

(1) $$\rho \left(S_{e}S+\frac{C_{m}}{\rho g}\right)\frac{\partial p}{\partial t}+\nabla \cdot \left(\rho u\right)=Q_{m}$$

(2) $$\rho \varepsilon_{p}=\theta_{s}$$

(3) $$u=-\frac{\kappa }{\mu }\nabla p$$

(4) $$\kappa =k_{s}k_{r}\left(S_{e}\right)$$

In the equation, ρ is the density of the solution; $S_{e}$ is the effective saturation; S is the specific storage coefficient; $C_{m}$ is the specific moisture capacity; p is the pore water pressure; g is the acceleration due to gravity; ∇ is the Hamiltonian operator; u is the flow velocity; $Q_{m}$ is the sink or source term of the solution; $\varepsilon_{p}$ is the porosity; $\theta_{s}$ is the saturation volumetric fraction; μ is the dynamic viscosity; κ is the permeability; $k_{s}$ is the saturated hydraulic conductivity; and $k_{r}$ is the relative hydraulic conductivity. The parameters related to unsaturated seepage in this model are all fitted using the Brooks–Corey model.

### 2.2. Transport of Dilute Species in Porous Media

The transfer of dilute species in porous media is mainly used to simulate the migration and reaction of substances in porous media, calculating the concentration and transfer of substances in porous media, including the transfer mechanisms of diffusion, convection, migration, dispersion, adsorption, and volatilization of chemical substances in porous media. The specific expression is as follows:

(5) $$\frac{\partial (\theta c_{i})}{\partial t}+\frac{\partial (\rho c_{P,i})}{\partial t}+\frac{\partial (\theta_{g}c_{G,i})}{\partial t}+\nabla \cdot J_{i}+\mu \cdot \nabla c_{i}=R_{i}+S_{i}$$

In the formula, θ is the volumetric water content; ρ is the density of the solution; $c_{i}$ is the solute concentration in the solution; $c_{P,i}$ is the adsorbed concentration in the solution; $c_{G,i}$ is the concentration of reaction products; $\theta_{g}$ is the liquid volume fraction; ∇ is the Hamiltonian operator; $\nabla \cdot J_{i}$ is the diffusion term of the equation, $J_{i}$ is the diffusion flux, where $J_{i}=-(D_{G,i}+D_{e,i})\nabla c_{i}$, $D_{G,i}$ is the dispersion tensor; $D_{e,i}$ is the effective diffusion coefficient; μ is the dynamic viscosity; $\mu \cdot \nabla c_{i}$ is the convective term of the equation; $R_{i}$ is the reaction term; $S_{i}$ is the sink or source term of the solution.

### 2.3. Reaction Kinetics Model

In the following sections, we will focus on the formulation of numerical prototypes for antigen–antibody reactions. These models are based on the following assumptions [26,27,28,29,30]:

- Antigens consist of single molecules and exist in homogeneous form, as do antibodies.
- One report particle combines one target analyte.
- Binding is consistent, without positive or negative allosteric effects (binding of one site on the analytes does not affect the binding of another site to the antibody).
- The reaction is a first-order reversible interaction, with concentrations of reactants reaching a steady state over time.
- There is no non-specific binding, such as binding to the reaction vessel walls.
- The rate constants of the reaction are constant, meaning they do not change with variations in reagent and sample concentrations during the reaction process.

#### 2.3.1. Sandwich LFIA Reaction Kinetics Model

The components of the model are represented as follows:

A—Analytes;

P—Reporter particle;

PA—Complex formed by analytes and reporter particle;

$R_{T}$—Capture probe, immobilized on the test line;

$R_{T}A$—Complex formed by analytes captured by the test line;

$R_{T}PA$—Complex formed by analytes, reporter particle, and antibody immobilized on the test line;

$R_{C}$—Capture probe, immobilized on the control line;

$R_{C}P$—Complex formed by free reporter particle captured by the control line;

$R_{C}PA$—Complex formed by analytes, reporter particle, and antibody immobilized on the control line.

**Figure 1:**
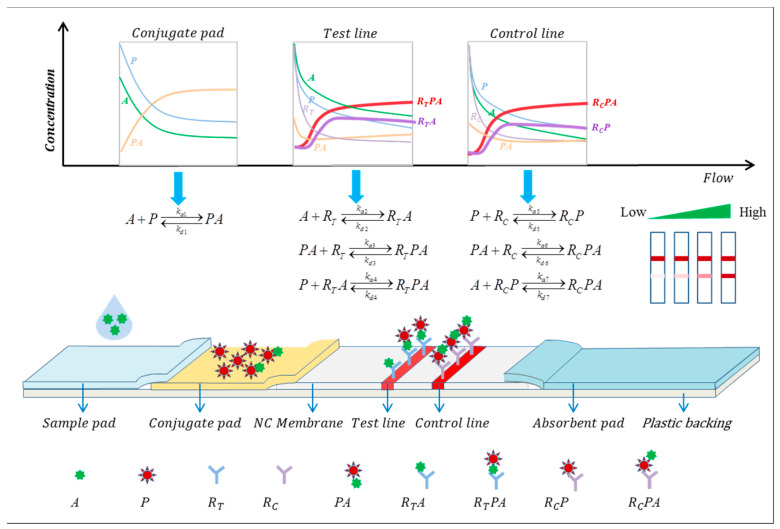
Schematic diagram of the sandwich LFIA model and reaction processes. The model depicts all the reaction processes involved in LFIA.

In the Sandwich LFIA reaction process and equations as depicted in Figure 1, the sample liquid is dispensed onto the sample pad, and due to capillary action, it migrates forward. The analytes (A) in the sample liquid reacts with the reporter particles (P) on the conjugate pad to form the analyte–reporter particle complex (PA) upon passing through the conjugate pad. When the mixture of (A), (P), and (PA) migrates to the test line (T-line), the capture antibody ($R_{T}$) on the test line captures the analytes (A) and the analyte–reporter particle complex (PA), forming ($R_{T}A$) and ($R_{T}PA$), respectively. The complex ($R_{T}A$) may further interact with free reporter particles (P) to form ($R_{T}PA$). At the control line, the reporter particles (P) and (PA) are captured by the control line antibody ($R_{C}$) to form ($R_{C}P$) and ($R_{C}PA$), respectively. The complex ($R_{C}P$) may further interact with free analytes (A) to form ($R_{C}PA$). These biochemical reactions are reversible, where $k_{ai}(i=1\sim 7)$ represent the association rate constants, and $k_{di}(i=1\sim 7)$ represent the dissociation rate constants.

During the reaction process, the concentrations of each substance can be considered as a function of the position x on the lateral flow strip and the reaction time t. They all satisfy the convection–diffusion equation and fluid dynamics equations. Solving these partial differential equations for the concentration functions of each substance at any position and time during the reaction process yields the concentration profiles. For ease of understanding, we represent each substance involved in the reaction with the letters or combinations of letters enclosed in parentheses. $c_{A},c_{P},c_{PA},c_{R_{T}},c_{R_{C}},c_{R_{T}A},c_{R_{T}PA},c_{R_{C}PA},c_{R_{C}P}$ denote the concentrations of the respective substances, with subscript 0 indicating the initial concentrations.

According to material conservation and reaction kinetics equilibrium, the reaction rates for each component are calculated as follows [26]:

(6) $$r_{PA}=k_{a1}c_{A}c_{P}-k_{d1}c_{PA}$$

(7) $$r_{R_{T}A}=k_{a2}c_{A}\left(c_{R_{T}0}-c_{R_{T}A}-c_{R_{T}PA}\right)-k_{d2}c_{R_{T}A}-k_{a4}c_{R_{T}A}c_{P}+k_{d4}c_{R_{T}PA}$$

(8) $$r_{R_{T}PA}^{1}=k_{a3}c_{PA}\left(c_{R_{T}0}-c_{R_{T}A}-c_{R_{T}PA}\right)-k_{d3}c_{R_{T}PA}$$

(9) $$r_{R_{T}PA}^{2}=k_{a4}c_{R_{T}A}c_{P}-k_{d4}c_{R_{T}PA}$$

(10) $$r_{R_{T}PA}=r_{R_{T}PA}^{1}+r_{R_{T}PA}^{2}$$

(11) $$r_{R_{C}P}=k_{a5}c_{P}\left(c_{R_{c}0}-c_{R_{C}P}-c_{R_{C}PA}\right)-k_{d5}c_{R_{C}P}-k_{a7}c_{R_{C}P}c_{A}+k_{d7}c_{R_{C}PA}$$

(12) $$r_{R_{C}PA}^{1}=k_{a6}c_{PA}\left(c_{R_{c}0}-c_{R_{C}P}-c_{R_{C}PA}\right)-k_{d6}c_{R_{C}PA}$$

(13) $$r_{R_{C}PA}^{2}=k_{a7}c_{R_{C}P}c_{A}-k_{d7}c_{R_{C}PA}$$

(14) $$r_{R_{C}PA}=r_{R_{C}PA}^{1}+r_{R_{C}PA}^{2}$$

The reaction rate constants for the analyte–antibody interaction are adopted from Qian [26], where $k_{a1}=k_{a2}=k_{a3}=k_{a4}=10^{6}\;M^{-1}s^{-1}$ and $k_{d1}=k_{d2}=k_{d3}=k_{d4}=10^{-3}\;s^{-1}$.

The diffusion–reaction equations for all species are as follows:

(15) $$\frac{\partial C_{i}}{\partial t}=D_{i}\frac{\partial C_{i}^{2}}{\partial x^{2}}-u\left(\frac{\partial C_{i}}{\partial x}\right)-r_{i}$$

where $c_{i}$ is the concentration of the species, $D_{i}$ is the diffusion coefficient, u is the fluid velocity, and $r_{i}$ is the reaction rate of species *i*. The complete set of equations for each species is listed as follows [26]:

(16) $$\frac{\partial c_{A}}{\partial t}=D_{A}\frac{\partial c_{A}^{2}}{\partial x^{2}}-u\left(\frac{\partial c_{A}}{\partial x}\right)-(r_{PA}+r_{R_{T}A}+r_{R_{C}PA}^{2})$$

(17) $$\frac{\partial c_{P}}{\partial t}=D_{P}\frac{\partial c_{P}^{2}}{\partial x^{2}}-u\left(\frac{\partial c_{P}}{\partial x}\right)-(r_{PA}+r_{R_{T}PA}^{2}+r_{R_{C}P})$$

(18) $$\frac{\partial c_{PA}}{\partial t}=D_{PA}\frac{\partial c_{PA}^{2}}{\partial x^{2}}-u\left(\frac{\partial c_{PA}}{\partial x}\right)+r_{PA}-r_{R_{T}PA}^{1}-r_{R_{C}PA}^{1}$$

(19) $$\frac{\partial c_{R_{T}A}}{\partial t}=r_{R_{T}A}$$

(20) $$\frac{\partial c_{R_{T}PA}}{\partial t}=r_{R_{T}PA}$$

(21) $$\frac{\partial c_{R_{C}P}}{\partial t}=r_{R_{C}P}$$

(22) $$\frac{\partial c_{R_{C}PA}}{\partial t}=r_{R_{C}PA}$$

where $D_{A}=10^{-10}\;m^{2}/s$ and $D_{P}{=D}_{PA}=10^{-12}\;m^{2}/s$ are the diffusion coefficients for the analytes (A) and the complex of analytes and reporter particles (PA), respectively [26].

#### 2.3.2. Competitive LFIA Reaction Kinetics Model

The commonly observed reaction mode in commercially available competitive LFIA involves the competition between the capture probe on the T-line and the antibody conjugated to the reporter particles for the target analytes. This study exclusively analyzes this competitive reaction model.

**Figure 2:**
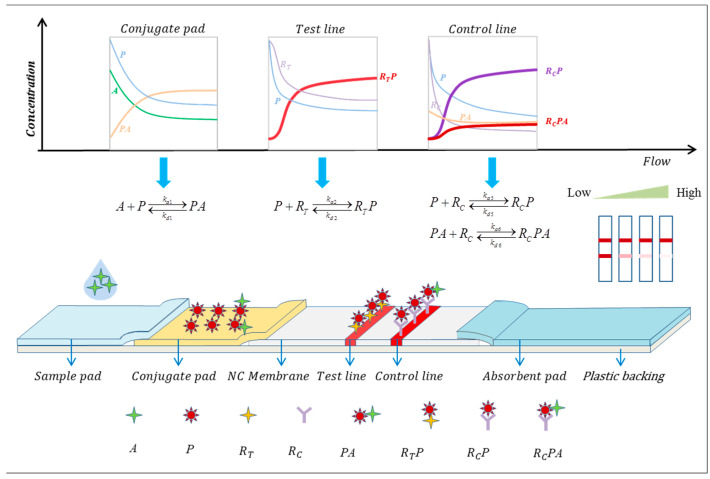
Schematic diagram of the competitive LFIA model and reaction processes. The model depicts all the reaction processes involved in LFIA.

The competitive LFIA reaction process and equations are shown in Figure 2. After the sample liquid is dropped onto the sample pad, due to the capillary action, the analytes (A) in the sample liquid reacts with the reporter particles (P) on the conjugate pad to form the analyte–reporter particle complex (PA) when passing through the conjugate pad. When the mixture of (A), (P), and (PA) migrates to the test line, the antigen analogue ($R_{T}$) on the test line captures the free reporter particles (P) to form ($R_{T}P$). At the control line, the reporter particles (P) and (PA) are captured by the control line antibodies ($R_{C}$) to form ($R_{C}P$) and ($R_{C}PA$), respectively. These biochemical reactions are reversible, where $k_{ai}(i=1\sim 4)$ represents the association rate constants, and $k_{di}(i=1\sim 4)$ represents the dissociation rate constants.

Assuming no time delay in the four biochemical reaction processes, the rates of the four biochemical reactions are given by [27]:

(23) $$r_{PA}=k_{a1}c_{A}c_{P}-k_{d1}c_{PA}$$

(24) $$r_{R_{T}P}=k_{a2}c_{P}\left(c_{R_{T}0}-c_{R_{T}P}\right)-k_{d2}c_{R_{T}P}$$

(25) $$r_{R_{C}PA}=k_{a3}c_{PA}\left(c_{R_{C}0}-c_{R_{C}PA}-c_{R_{C}P}\right)-k_{d3}c_{R_{C}PA}$$

(26) $$r_{R_{C}P}=k_{a4}c_{P}\left(c_{R_{C}0}-c_{R_{C}PA}-c_{R_{C}P}\right)-k_{d4}c_{R_{C}P}$$

From the convection–diffusion equations, we can obtain:

(27) $$\frac{\partial c_{A}}{\partial t}=D_{A}\frac{\partial c_{A}^{2}}{\partial x^{2}}-u\left(\frac{\partial c_{A}}{\partial x}\right)-r_{PA}$$

(28) $$\frac{\partial c_{P}}{\partial t}=D_{P}\frac{\partial c_{P}^{2}}{\partial x^{2}}-u\left(\frac{\partial c_{P}}{\partial x}\right)-{(r}_{PA}+r_{R_{T}P}+r_{R_{C}P})$$

(29) $$\frac{\partial c_{PA}}{\partial t}=D_{PA}\frac{\partial c_{PA}^{2}}{\partial x^{2}}-u\left(\frac{\partial c_{PA}}{\partial x}\right)+r_{PA}-r_{R_{C}PA}$$

(30) $$\frac{\partial c_{R_{T}P}}{\partial t}=r_{R_{T}P}$$

(31) $$\frac{\partial c_{R_{C}P}}{\partial t}=r_{R_{C}P}$$

(32) $$\frac{\partial c_{R_{C}PA}}{\partial t}=r_{R_{C}PA}$$

## 3. Finite Element Simulation

### 3.1. Simulation Approach

**Figure 3:**
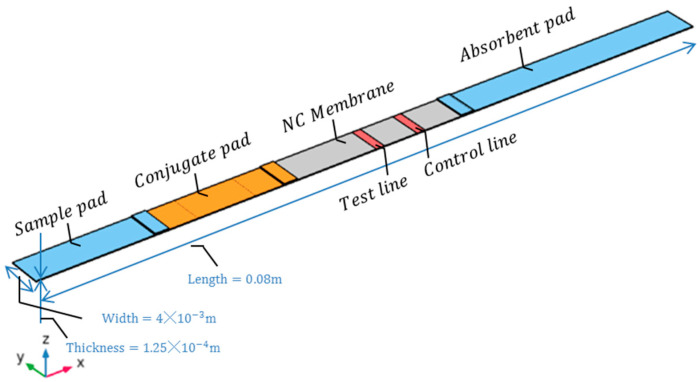
Schematic diagram of the 3D geometry model of LFIA.

**Figure 4:**
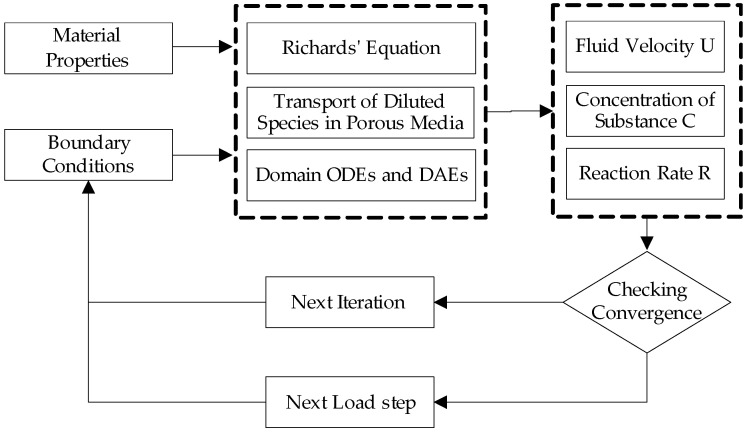
Flowchart of the solution scheme for LFIA simulation.

In COMSOL, LFIA three-dimensional models were established using the Richards equation, transport of dilute species in porous media, and domain ordinary differential equation physics interfaces, and the geometric diagram of the model is shown in Figure 3. The simulation process involves iterative calculations of fluid velocity, substance concentration distribution, and reaction rate coupling fields. During the solving process, COMSOL utilizes iterative algorithms to gradually approximate the solution of the model, as illustrated in Figure 4.

Set material properties and initial conditions for the model, including porous media and fluid. Specifically, materials such as NC membrane and other components (sample pad, conjugate pad, absorbent pad) are set as porous materials with different parameters (porosity, pore size, permeability, etc.), as detailed in Table 1. The initial conditions of the model include the initial concentration distribution of various substances, the initial state of the porous media, and the initial conditions of the domain, as described in Section 3.2. Select the solver and iterative algorithm to start iterative calculations. In each iteration, COMSOL updates boundary conditions based on the current solution and adjusts them according to residuals or other convergence criteria. Using the updated boundary conditions, the solver is called again, gradually approaching the final solution. This iteration scheme is repeated until the fluid velocity becomes zero, but the reaction–diffusion process continues to evolve. The solver continues to iterate until the final time step is reached.

### 3.2. Model Parameter Settings, Initial Conditions, and Boundary Conditions

Set model parameters, including geometric parameters, porous material parameters, physical parameters of the Richards equation and initial concentrations of each substance. The main model parameters include the following:

Using the Richards equation to model liquid-phase transport in porous media, the following are defined:

No-flow boundary:

(33) −n⋅ρu=0

Initial pressure value:

(34)  p=p0

Sample pad as fluid inlet, pressure head:

(35) $$H_{p0}=p0/(\rho g)+(Hw-p0/(\rho g))$$

The solving substance concentration and reaction rate are determined using the transport of dilute species in porous media and domain ODEs and DAEs interfaces. Fluid velocity is determined by the Richards equation, selecting the corresponding reaction region, setting the initial substance concentration as:

(36) $$c_{P}={c_{R_{T}}=c_{R_{C}}=c}_{0}$$

Initial concentration of other substances is set to 0.

(37) $$\begin{array}{c} c_{i}=0 \end{array}$$

The sample pad is set as the inlet, with the inlet boundary condition:

(38) $$c_{A}=c_{0}$$

The absorbent pad is the outlet, with the outlet boundary condition:

(39) $$n\cdot D_{i}\nabla c_{i}=0$$

The reaction rate of each substance in the corresponding reaction region is set, with the sandwich LFIA according to Equations (5)–(13) and the competitive LFIA according to Equations (22)–(25).

### 3.3. Parameter Definitions

When the computing system reaches equilibrium, the detection signal is obtained at 600 s. Theoretically, the concentration of the composite particles captured on the test line and control line is proportional to the available detection signal (optical signal, magnetic signal) in experiments. Therefore, in this study, the volume-averaged concentrations of the composite particles captured on the T-line and C-line are defined as the T-line and C-line analysis signals:

Sandwich LFIA:

(40) $$\;S_{T}={∭}_{V}c_{R_{T}PA}\frac{dV}{V}$$

(41) $$S_{C}={∭}_{V}(c_{R_{C}PA}+c_{R_{C}P})\frac{dV}{V}$$

where *V* is the volume of the T-line or C-line.

In sandwich LFIA, we take 1.5 × 10^−9^ M as the threshold value S_TL_ for S_T_. The target analyte concentration (C_AL_) corresponding to the threshold value S_TL_ is defined as the detection limit (C_AL_). The range of target analyte concentrations (C_AM_) corresponding to the maximum signal (S_Tmax_) from the detection limit (C_AL_) to S_T_ is defined as the working range (WR) [32]:

(42) $$WR=log\left(C_{AM}\right)-log(C_{AL})$$

Competitive LFIA:

(43) $$S_{T}={∭}_{V}c_{R_{T}P}\frac{dV}{V}$$

(44) $$S_{C}={∭}_{V}(c_{R_{C}PA}+c_{R_{C}P})\frac{dV}{V}$$

In Competitive LFIA, the detection limit for competitive LFIA is chosen as IC10, and the working range is IC20–IC80.

## 4. Results and discussion

### 4.1. The Effect of Flow Velocity on LFIA Performance

Flow velocity is one of the most important factors affecting the sensitivity of LFIA. In LFIA, once the analytes and reporter particles in the sample solution pass through the T-line and C-line, they cannot be captured. A slower flow velocity allows more interactions between reporter particles and capture reagents, leading to higher signal intensity [36]. Flow velocity is difficult to measure accurately in experiments, but it can be easily calculated in finite element simulation software.

**Figure 5:**
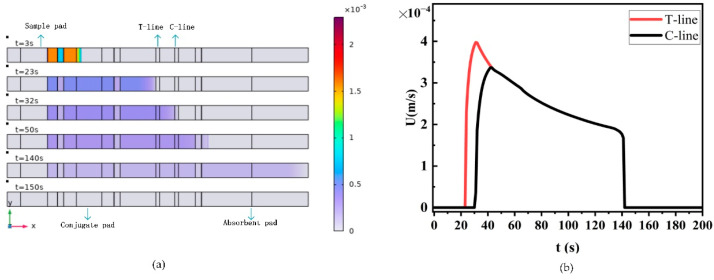
Sample liquid flow velocity figure. (**a**) Sample flow velocity contour plot; (**b**) flow velocity plot at T-line and C-line positions.

We simulated the flow velocity on the test strip using the Richards equation (Figure 5). The distribution of sample liquid flow velocity at different times on the test strip is shown in Figure 5a. The sample solution reaches the binding pad, T-line, C-line, and absorption pad at 3 s, 23 s, 32 s, and 50 s, respectively, and fills the entire test strip by 140 s, with the flow velocity rapidly decreasing to 0.

For comparison, we extracted the flow velocity profiles at the T-line and C-line (Figure 5b). It can be observed that the sample flow velocity decreases almost exponentially as the distance of the liquid front from the origin increases. This is because the capillary forces driving liquid flow only acts on the surface of the porous material where the sample solution meets the air (the three-phase boundary region of the liquid front). This means that the capillary force is constant, and as long as there is free pore volume, it can be filled with the sample solution. However, as the sample solution further enters the test strip, the flow resistance increases, leading to a decrease in flow velocity. The trend of the results calculated by the model is consistent with the trend derived by Mendez [37] based on the Lucas–Washburn equation.

**Figure 6:**
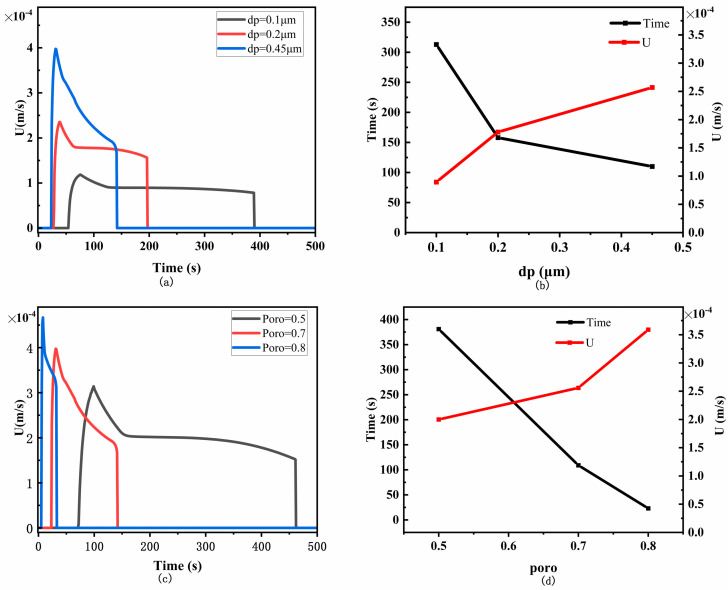
Effect of different pore sizes and porosities on flow velocity. (**a**) Flow velocity of liquid passing through the T-line for different pore sizes; (**b**) time and average velocity of liquid passing through the T-line for different pore sizes; (**c**) flow velocity of liquid passing through the T-line for different porosities; (**d**) time and average velocity of liquid passing through the T-line for different porosities.

The flow velocity of the membrane mainly depends on the properties of the porous structure, including pore size and porosity, and discussing these parameters helps us to understand capillary flow. Flow velocity is difficult to measure accurately in experiments, so commercial NC membranes typically use a parameter called capillary flow time (CFT) to reflect capillary flow velocity—the time required for the liquid to move along a specified length of strip and completely fill it. The larger the CFT value, the slower the capillary flow. We compared the flow velocities of liquids with different pore sizes and porosities passing through the T-line, and extracted the time and average velocity at which the liquid passes through the T-line (Figure 6).

Pore size is a measure of the maximum pore diameter. In the simulated experiments, we compared three common pore sizes (dp = 0.1, 0.2, 0.45 μm) found in the market and plotted the flow velocity of the liquid passing through the T-line at different pore sizes (Figure 6a). From the graph, it can be observed that as the pore size increases, the membrane’s flow velocity also increases. For easier observation, we extracted the time taken for the liquid to flow through the T-line from Figure 6a and calculated its average velocity (Figure 6b). It is evident that with an increase in pore size, the flow velocity of the membrane increases, and the time taken for the liquid to flow through the T-line decreases.

Porosity is the volume of air in a three-dimensional membrane–membrane structure, usually expressed as a percentage of the total membrane volume. Porosity is typically unrelated to pore size and is not controlled by pore size. These two parameters are essentially independent. In the simulation, we set the porosity to 0.5, 0.7, and 0.8, and compared the flow velocity of the liquid passing through the T-line (Figure 6c). It can be observed that under the same conditions, the capillary flow velocity increases with an increase in porosity, and at the same time, the time taken for the liquid to flow through the T-line decreases (Figure 6d). When the porosity is set to 0.7, the simulated flow velocity of the liquid through the T-line is 0.00025 m/s, which is close to the average flow velocity of 0.0002 m/s measured in the experiments by Qian [26,27] and Liu [31]. This validates the accuracy of the model.

**Figure 7:**
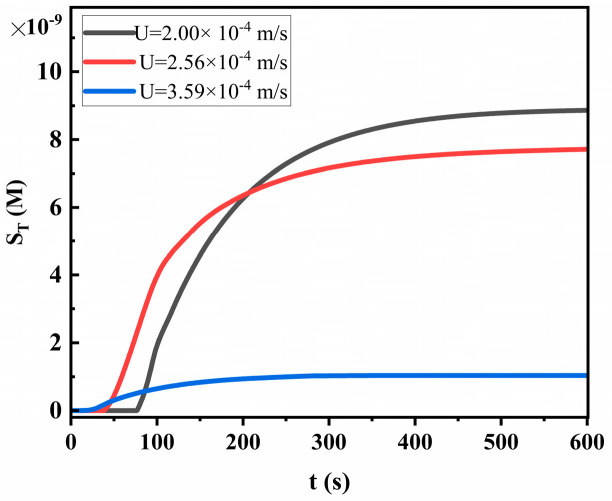
The trend of S_T_ under different flow velocities.

For a better observation of the impact of flow velocity on LFIA performance, we compared the variation trend of S_T_ (taking competitive LFIA as an example) under different average flow velocities (Figure 7). As the flow velocity increases, the time for the liquid to flow on the T-line decreases, shortening the time for the reactants to come close enough to bind, significantly reducing S_T_ and system sensitivity. This is why membranes with the fastest capillary flow are usually not used. The longer the reaction duration in the T-line and C-line, the higher the detection sensitivity and signal intensity of the T-line and C-line. Therefore, to achieve better analytical characteristics, the fluid front must pass through the T-line and C-line as slowly as possible. The structure of the NC membrane can be appropriately optimized (by selecting smaller pore size and lower porosity) or the viscosity of the sample can be increased to achieve this effect.

### 4.2. Sandwich LFIA

#### 4.2.1. Sandwich LFIA Reaction Process

**Figure 8:**
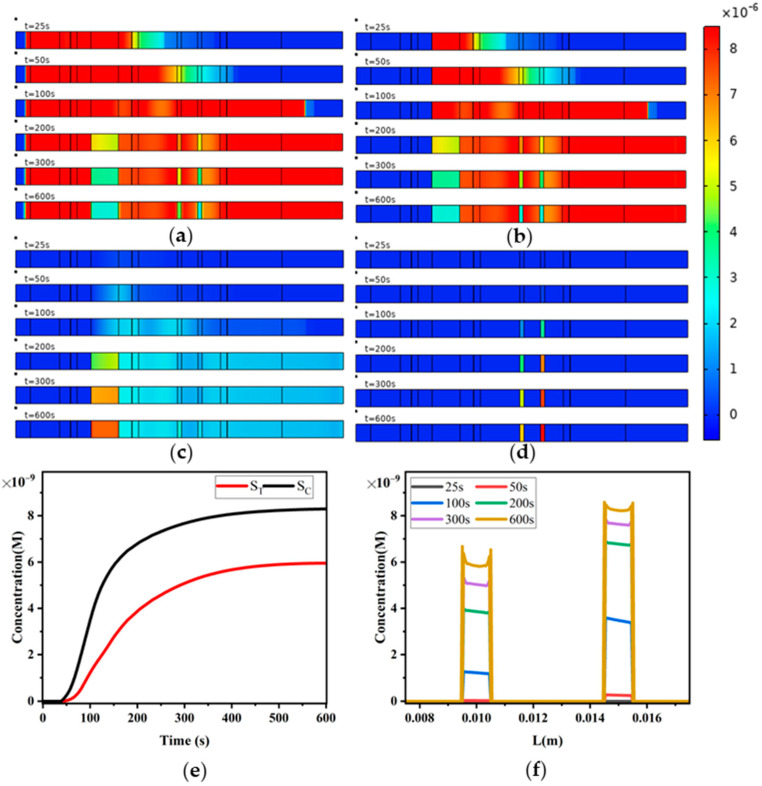
Sandwich LFIA reaction process. (**a**) Concentration distribution map of analytes A; (**b**) concentration distribution map of reporting particles P; (**c**) concentration distribution map of complex PA formed by reporting particles and analytes; (**d**) concentration distribution map of reporting particle complexes captured by T-line and C-line; (**e**) variation in signals S_T_ and S_C_ over time for T-line and C-line; (**f**) diffusion effects of reporting particle complexes captured by T-line and C-line.

In this section, we explore the reaction process of the sandwich LFIA under the non-uniform flow conditions calculated in the previous section. The model assumes that each reaction component is uniformly distributed throughout the thickness of the entire test strip, and here, the model’s top view is used to represent the calculation results. Figure 8 shows the concentration distribution of analytes A (Figure 8a), reporter particles P (Figure 8b), analyte–reporter particle complex PA (Figure 8c), complexes R_T_PA, R_C_PA, R_C_P formed by capturing reporter particles on the T-line and capturing PA and reporter particles P on the C-line (Figure 8d) at different times.

From Figure 8a, it can be observed that the analytes A, under the influence of capillary forces, exhibit lower concentration at the leading edge of the liquid flow. As it flows towards the right end of the absorbent pad, analytes A fill the entire test strip. A is consumed in the reaction zones including the binding pad, T-line, and C-line, leading to a decrease in concentration, while it is almost uniformly distributed elsewhere on the strip. The concentration distribution of reporting particles P (Figure 8b) is similar to that of the analytes A concentration distribution.

In Figure 8c, it can be seen that the complex PA travels with the liquid flow until it reaches the regions of T-line and C-line. Here, it reacts with the capture probes R_T_ and R_C_ on the T-line and C-line, respectively, forming colored detection lines (Figure 8d).

To further facilitate the observation of the reaction process at T-line and C-line, we analyzed the variation in S_T_ and S_C_ over time (Figure 8e). When the sample liquid reaches the T-line and C-line, particle concentrations begin to increase. As PA is captured in the regions of T-line and C-line, forming detection materials R_T_PA and R_C_PA, S_T_ and S_C_ exhibit nearly linear growth. When the absorbent pad saturates and flow stops, the growth in concentration slows down, indicating that under this condition, the formation of R_T_PA and R_C_PA is controlled by mass transport. Figure 8f displays the concentration distribution of the generated species along the x-direction on the surfaces of T-line and C-line, showing edge effects. The concentration is higher near x = 0, which can be easily explained as this edge is closest to the inlet where reactants are first captured. At the edge near the outlet, the concentration also increases due to diffusion effects, and this effect is also present at the edge positions near the inlet.

#### 4.2.2. The Influence of Target Analyte Concentration on S_T_ and S_C_ in Sandwich LFIA 

**Figure 9:**
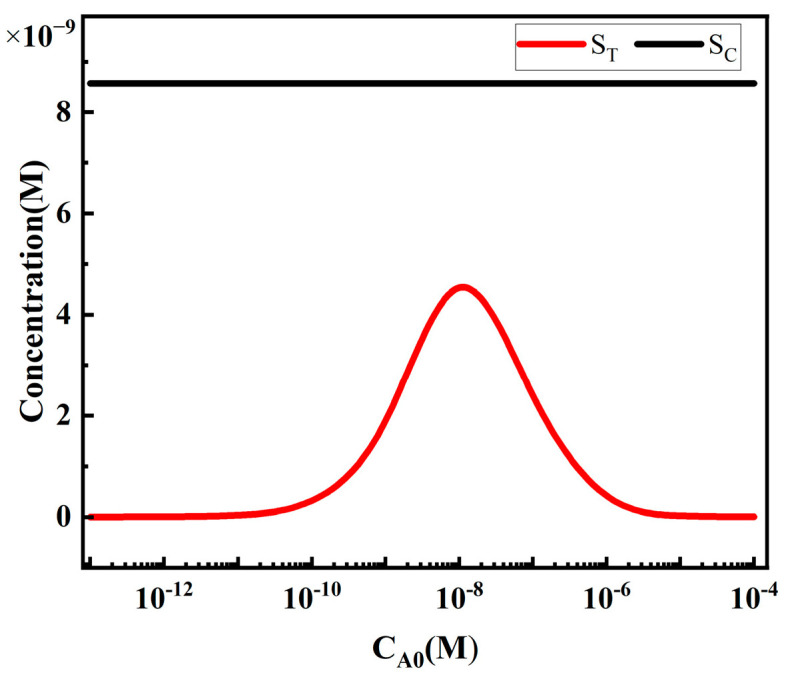
The variation in S_T_ and S_C_ with the concentration of analytes.

Figure 9 depicts the variation in S_T_ and S_C_ with the concentration of analytes A. When the concentration of the target analytes is very low, S_T_ remains nearly constant. As the concentration of the target analytes increases, S_T_ almost linearly increases as a function of C_A0_. With further increase in the concentration of the target analytes, S_T_ reaches its maximum value at the concentration C_AM_ of the target analytes, and then decreases. C_AM_ corresponds to the concentration of the target analytes at the peak of S_T_, a phenomenon known as the “HOOK effect”, which is described in many literature sources. At high concentrations, the decrease in S_T_ is due to the binding of analytes to reporting particles and capture probes, which hinders the binding of complex PA to R_T_. S_C_ remains almost constant as the concentration of analytes changes, indicating that the concentration of analytes does not affect S_C_.

#### 4.2.3. The Influence of Reporter Particle Concentration on Sandwich LFIA Performance

**Figure 10:**
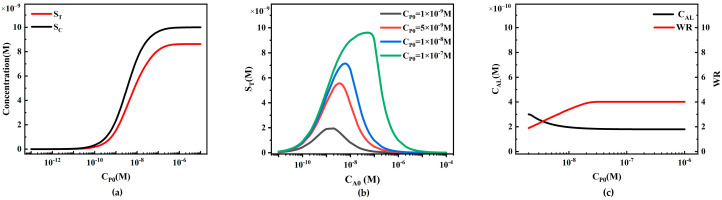
The relationship between reporter particle concentration and LFIA detection performance. (**a**) Variation in S_T_ and S_C_ with different reporter particle concentrations; (**b**) standard curves of the HOOK effect under different reporter particle concentrations; (**c**) trends of LFIA detection limit (C_AL_) and working range (WR) under different reporter particle concentrations.

The concentration of reporter particles P plays a significant role in the sensitivity of LFIA. We first investigated the effect of reporter particle concentration on the performance of sandwich LFIA (Figure 10).

When the analyte concentration remains constant, an increase in the concentration of reporter particles P positively affects the signal intensity on the T-line and C-line (Figure 10a). The simulation results show that at low concentrations, C_P0_ is low, and may not sufficiently cover the binding sites on the test and control lines, resulting in relatively constant concentrations of S_T_ and S_C_. As C_P0_ concentration increases, more binding sites are occupied, and the concentrations of S_T_ and S_C_ begin to increase almost linearly. However, when C_P0_ concentration further increases to a certain extent, the binding sites on the test and control lines may become saturated, leading to a slowing down of the rate of increase in concentrations of S_T_ and S_C_, showing a saturation state.

We also plotted standard curves of the HOOK effect for different C_P0_ values (1 × 10^−9^, 5 × 10^−9^, 1 × 10^−8^, and 1 × 10^−7^ M) (Figure 10b). The increase in initial concentration of reporter particles not only enhances the S_T_ value corresponding to the peaks in the standard curve, but also results in a rightward shift in the peak in the HOOK effect standard curve. The rightward shift indicates an increase in the working range, possibly due to the increased strength of binding between reporter particles and analytes.

To further compare LFIA performance, we extracted the detection limit (C_AL_) and working range (WR) from the simulation results (Figure 10c). With the increase in CP0, the C_AL_ in the simulation shows a decreasing trend (black line in Figure 10c), indicating that higher C_P0_ enhances system sensitivity. However, due to the limited capture capacity of the capture probes fixed on the T-line and C-line, this decreasing trend gradually becomes moderate. Additionally, it can be observed from the simulation results that as C_P0_ increases, the WR widens (red line in Figure 10c), indicating that appropriately increasing C_P0_ is beneficial for expanding the linear working range. The simulation results also reveal that the slopes of the C_AL_ and WR curves gradually decrease, indicating that the capture capacity of the capture reagents on the T-line and C-line gradually saturates, and cannot bind more reporter particles. This model can be utilized to predict the standard curves, C_AL_, and WR under different C_P0_ values, assisting experimenters in selecting the optimal C_P0_ for LFIA with the best performance.

#### 4.2.4. The Influence of Initial Capture Probe Concentration on Sandwich LFIA Performance

**Figure 11:**
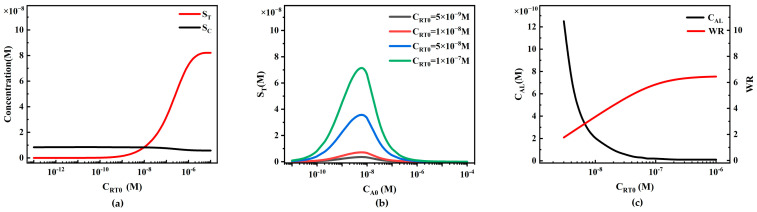
The relationship between the initial capture probe concentration on the T-line and LFIA detection performance. (**a**) Variations in S_T_ and S_C_ with different initial capture probe concentrations on the T-line; (**b**) standard curves of the HOOK effect under different initial capture probe concentrations on the T-line; (**c**) trends of LFIA detection limit (C_AL_) and working range (WR) under different initial capture probe concentrations on the T-line.

The initial concentration of capture probe C_RT0_ on the T-line is also a crucial parameter when preparing LFIA, and we investigated its impact on LFIA performance by calculating different concentrations of C_RT0_ (Figure 11).

When other conditions remain constant, C_RT0_ only affects S_T_, with little influence on S_C_ (Figure 11a). When C_RT0_ is low, the effective capture of reporter particles and analytes is insufficient, resulting in a low and relatively constant S_T_. As the concentration of capture antibodies increases, the capture capacity increases, leading to an almost linear increase in S_T_. However, when the binding sites of the capture antibodies reach saturation (due to the limitation of C_P0_, preventing further binding of more reporter particles), even with further increases in the concentration of capture antibodies, S_T_ cannot be further increased, reaching a saturation state. In the range of C_RT0_ = 1 × 10^−8^ to 1 × 10^−6^ M, when S_T_ increases almost linearly, more reporter particles P are captured on the T-line, resulting in a decrease in reporter particles P on the downstream C-line, thus reducing S_C_.

We plotted concentration curves of S_T_ at different analyte concentrations using different C_RT0_ values (5 × 10^−9^, 1 × 10^−8^, 5 × 10^−8^, and 1 × 10^−7^ M) (Figure 11b). The increase in C_RT0_ enhances the capture capacity of the T-line, enlarging the peak of the S_T_ curve, significantly reducing the detection limit. As C_RT0_ increases, the C_AL_ in the simulation shows a decreasing trend (black line in Figure 11c), indicating that higher C_RT0_ also increases sensitivity. However, as C_RT0_ increases, this decreasing trend gradually becomes less sensitive. The changes in the detection limit with different C_RT0_ are more pronounced compared to different C_P0_. Additionally, the WR in the simulation shows an expanding trend with the increase in capture probe concentration (red line in Figure 11c). Therefore, increasing C_RT0_ should be considered to lower the system detection limit and expand the system detection range. Similarly, this model can be used to predict standard curves, C_AL_, and WR under different C_RT0_ values, helping experimenters select the optimal C_RT0_ for LFIA with the best performance.

**Figure 12:**
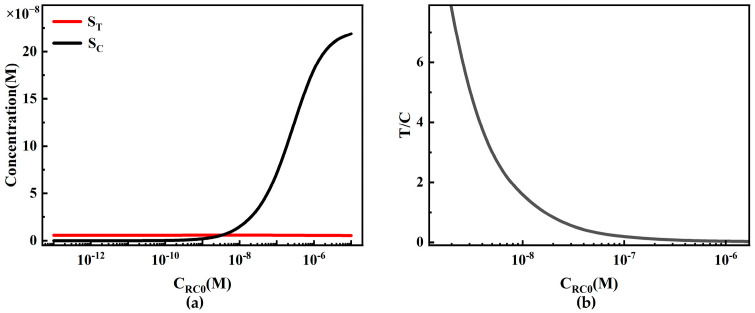
The relationship between the initial capture probe concentration of the C-line and the LFIA detection performance. (**a**) The variation in S_T_ and S_C_ under different initial capture probe concentrations of the C-line; (**b**) the trend of T/C under different initial capture probe concentrations of the C-line.

We also studied the influence of the initial concentration of capture probe C_RC0_ on LFIA performance (Figure 12). Theoretically, C_RC0_ only affects S_C_, with little influence on S_T_ (Figure 12a). When C_RC0_ is low, the effective capture of reporter particles and analyte–reporter particle complexes is insufficient, resulting in a low and relatively constant S_C_. As the concentration of capture antibodies increases, the capture capacity increases, leading to an almost linear increase in S_C_. However, as C_RC0_ continues to increase, the trend of an increase in S_C_ slows down due to the limitation of C_P0_, preventing further binding of more reporter particles.

Because S_T_ is insensitive to changes in C_RC0_, similarly, the system detection limit and working range are also insensitive to changes in C_RC0_. However, in commercial quantitative detection, to reduce inter-batch variability, the T/C value is usually used as the final detection signal. When S_C_ increases linearly with C_RC0_, the T/C value (S_T_/S_C_) decreases (Figure 12b). In practical detection, this change may lead to a decrease in detection sensitivity due to the influence of detection noise. Therefore, in experiments, C_RC0_ should be limited to avoid adversely affecting the system detection performance by keeping the T/C value from being too low.

#### 4.2.5. The Influence of Reaction Rate Constants on Sandwich LFIA Performance

**Figure 13:**
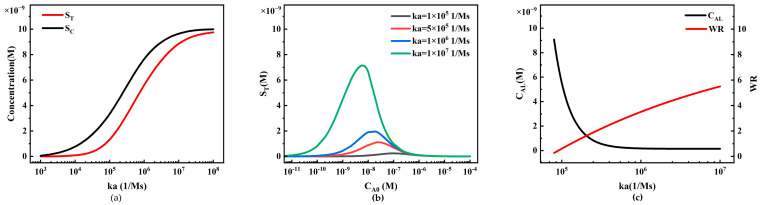
The relationship between the association rate constant *ka* and LFIA detection performance. (**a**) Variation in S_T_ and S_C_ under different association rate constants *ka*; (**b**) standard curves of the HOOK effect under different association rate constants *ka*; (**c**) trends in LFIA detection limits and working ranges under different association rate constants *ka*.

**Figure 14:**
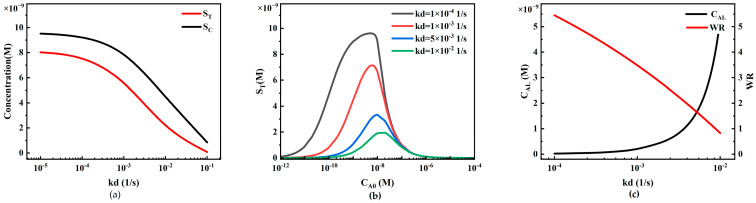
The relationship between the dissociation rate constant *kd* and LFIA detection performance. (**a**) Variations in S_T_ and S_C_ under different dissociation rate constants *kd*; (**b**) standard curves of the HOOK effect under different dissociation rate constants *kd*; (**c**) trends in LFIA detection limits and working ranges under different dissociation rate constants *kd*.

The reaction rate constants, including the association rate constant *ka* and the dissociation rate constant *kd*, play a crucial role in LFIA performance. Significant effort is required in experimental research to select antibodies with different *ka* and *kd* values because different *ka* and *kd* values correspond to different reagent reactions in various LFIA detection processes. Therefore, we can utilize simulation methods to analyze the impact of *ka* and *kd* values on antigen–antibody binding reactions (Figure 13 and Figure 14).

From Figure 13a, we observe the variations in S_T_ and S_C_ under different association rate constants *ka* at constant analyte concentrations. A larger *ka* implies improved reaction efficiency of the reagents, resulting in higher S_T_ and S_C_ at the T-line and C-line. However, when the binding sites of the capture probes on the T-line and C-line become saturated, S_T_ and S_C_ tend to saturate as well.

In Figure 13b, we observe the phenomenon of the HOOK effect under different association rate constants (*ka* = 1 × 10^5^, 5 × 10^5^, 1 × 10^6^, 1 × 10^7^ M^−1^ s^−1^). An increase in *ka* significantly shifts the peak of the HOOK curve to the left (lower analyte concentrations). From Figure 13b, we extract the system detection limit (C_AL_) and working range (WR) (Figure 13c). With an increase in *ka*, the detection limit decreases significantly, and the working range gradually expands. This is mainly because higher *ka* enhances the reaction efficiency of the reagents, leading to greater capture efficiency of the capture probes on the test line and stronger binding capacity.

The impact of the dissociation rate constant *kd* on system performance is exactly the opposite of the association rate constant *ka* (Figure 14). An increase in *kd* leads to a decrease in the reaction rate, significantly reducing the capture capacity of the T-line and C-line, resulting in a decrease in S_T_ and S_C_ (Figure 14a). In the HOOK effect curves at different analyte concentrations (Figure 14b), an increase in *kd* leads to a rightward shift in the curve peak, indicating an increase in the detection limit, which reduces the sensitivity of the system, while the working range also narrows (Figure 14c).

These phenomena indicate that increasing the association rate *ka* and decreasing the dissociation rate *kd* are beneficial for quantitative analysis in rapid LFIA detection. By selecting antibodies or capture probes with higher association rate constants and lower dissociation rate constants, we can optimize the performance of LFIA detection. However, it is important to note that antibodies or capture probes with higher association rates may be accompanied by higher dissociation rates. Therefore, when designing LFIA, a balanced consideration between association and dissociation rates is needed to achieve optimal performance.

### 4.3. Competitive LFIA

#### 4.3.1. Competitive LFIA Reaction Process

**Figure 15:**
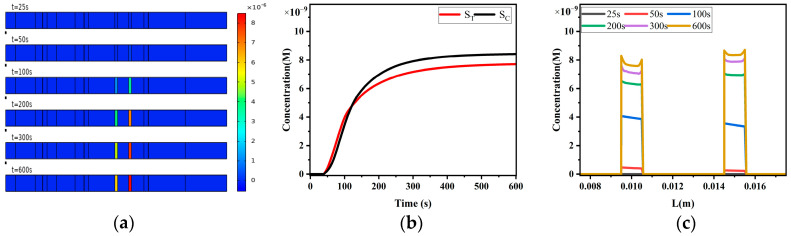
Competitive LFIA reaction process. (**a**) Concentration distribution map of reporter particle complexes captured by T-line and C-line; (**b**) variation in T-line and C-line signals S_T_ and S_C_ over time; (**c**) diffusion effects of reporter particle complexes captured by T-line and C-line.

Similarly to the sandwich LFIA, we explored the reaction process of competitive LFIA under non-uniform fluid flow conditions (Figure 15). The concentration distribution of analytes A, reporter particles P, and analyte–reporter particle complexes PA in competitive LFIA is similar to that in sandwich LFIA, and will not be repeated here. The concentration distribution of complexes RTP captured by the T-line, PA captured by the C-line, and complexes R_C_PA and R_C_P formed by PA and P capture is shown in Figure 15a. Under the same conditions, the color of the T-line in competitive LFIA is darker. This is because in sandwich LFIA, part of the T-line capture probe reacts with A in the analyte, resulting in a reduction in binding sites and a decrease in the capture capability for free reporter particles P.

Figure 15b depicts the variation in T-line and C-line signals S_T_ and S_C_ over time. According to the reaction kinetics equation, the T-line only captures free reporter particles P, while the C-line captures not only free reporter particles P but also the reporter particle–analyte complex PA. Therefore, after the reaction reaches equilibrium, S_C_ is higher than S_T_.

Figure 15c describes the distribution of product concentration along the x-direction on the surfaces of the T-line and C-line. Similarly to sandwich LFIA, competitive LFIA exhibits edge effects in the concentration distribution along the surfaces of the T-line and C-line, with stronger signals at the edges of the T-line and C-line.

#### 4.3.2. The Influence of Target Analyte Concentration on S_T_ and S_C_ in Competitive LFIA

**Figure 16:**
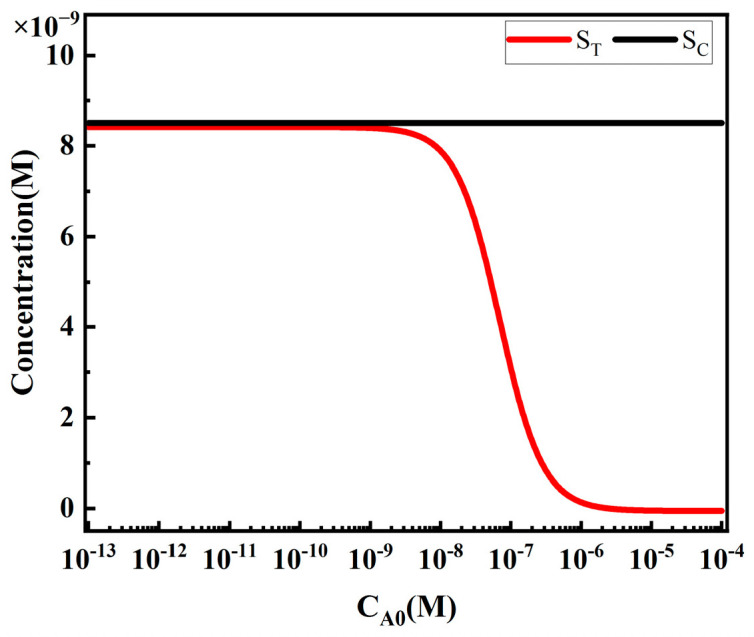
Impact of target analyte concentration on S_T_ and S_C_.

The influence of target analyte concentration on S_T_ follows a sigmoidal curve (Figure 16), which is a typical form of calibration curves in competitive analysis [30]. When the target analyte concentration is low, the analytes A have little effect on the concentration of free reporter particles P, resulting in a high and relatively constant S_T_ value. As the analyte concentration increases, the competitive reaction between analytes A and reporter particles P intensifies, leading to a decrease in the concentration of free reporter particles P and a nearly linear decrease in S_T_. When the analyte concentration reaches a certain level, the competitive reaction between analytes A and reporter particles P saturates, and S_T_ no longer changes with increasing analyte concentration. S_C_, on the other hand, remains unaffected by changes in analyte concentration, which is why the T/C ratio is commonly used as the detection result in commercial assays.

#### 4.3.3. The Influence of Reporter Particle Concentration on Competitive LFIA Performance

**Figure 17:**
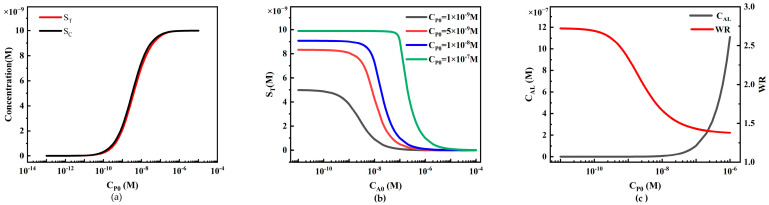
The relationship between reporter particle concentration and LFIA detection performance. (**a**) Variations in S_T_ and S_C_ under different reporter particle concentrations. (**b**) Inverse S-shaped standard curves at different reporter particle concentrations. (**c**) Trends of LFIA detection limits and working ranges under different reporter particle concentrations.

Similarly to the sandwich LFIA, we first analyze the effect of reporter particle concentration on the performance of competitive LFIA (Figure 17). Under constant analyte concentration conditions, the trends of S_T_ and S_C_ at different reporter particle concentrations are similar to those in the sandwich LFIA. At low C_P0_ concentrations, the changes in S_T_ and S_C_ are minimal. As C_P0_ increases, both S_T_ and S_C_ linearly depend on C_P0_. Followed by a slower growth rate, this is due to the saturation of binding sites for capture probes on the T-line and C-line.

We also plotted inverse S-shaped standard curves for different C_P0_ values (1 × 10^−9^, 5 × 10^−9^, 1 × 10^−8^, and 1 × 10^−7^ M) (Figure 17b). The increase in C_P0_ results in a rightward shift in the curve, with both detection limit and working range moving towards higher analyte concentrations. This is because as C_P0_ increases, more reporter particles can bind to more target analytes to form reporter particle–analyte complexes PA. However, due to competitive effects, this hinders the recognition and capture of reporter particles by the T-line capture probes.

To further compare LFIA performance, we extracted detection limits (C_AL_) and working ranges (WR) from simulation results (Figure 17c). As C_P0_ increases, C_AL_ shows an upward trend in the simulation (black line in Figure 17c), indicating that higher C_P0_ actually decreases system sensitivity, with this effect becoming more pronounced at higher concentrations. Additionally, it can be observed from the simulation results that as C_P0_ increases, WR narrows (red line in Figure 17c). Unlike the sandwich LFIA, an increase in C_P0_ actually raises the system detection limit and narrows the working range, consistent with the conclusion in reference [30]. This suggests that higher C_P0_ concentrations increase the detection signal strength, but reducing C_P0_ can be used to enhance the detection sensitivity of competitive LFIA. We can utilize this model to predict standard curves, C_AL_, and WR under different C_P0_ values and assist experimenters in selecting the optimal C_P0_ for LFIA performance.

#### 4.3.4. The Influence of Capture Probe Initial Concentration on Competitive LFIA Performance

**Figure 18:**
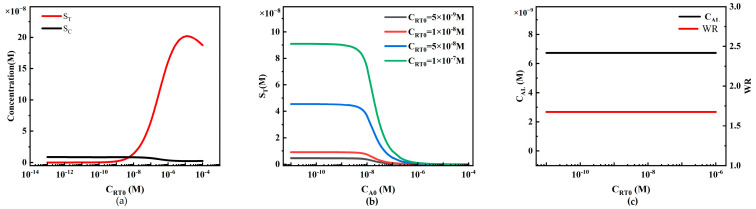
The Relationship between T-Line Capture Probe Initial Concentration and LFIA Detection Performance: (**a**) Variations in S_T_ and S_C_ under Different T-Line Capture Probe Initial Concentrations; (**b**) Inverse S-shaped Standard Curves under Different T-Line Capture Probe Initial Concentrations; (**c**) Trends of LFIA Detection Limits and WR under Different T-Line Capture Probe Initial Concentrations.

Similarly to the sandwich LFIA, the initial concentration of the T-line capture probe, C_RT0_, only affects S_T_ and has minimal impact on S_C_ (Figure 18a). When C_RT0_ is low, it fails to effectively capture reporter particles and analytes, resulting in lower and relatively unchanged S_T_. As the concentration of capture antibodies increases, the capturing capability enhances, leading to an almost linear increase in S_T_. However, at high C_RT0_ concentrations, S_T_ decreases instead. This might be attributed to the limited binding capacity of proteins on the membrane, causing protein accumulation and spatial hindrance effects when C_RT0_ concentration is too high, inhibiting the capture reaction of reporter particles P. As S_T_ almost linearly increases, more reporter particles P are captured by the T-line, reducing the concentration of reporter particles C_P0_ downstream of the T-line and thus lowering S_C_.

We plotted inverse S-shaped curves for S_T_ at different analyte concentrations using different C_RT0_ values (5 × 10^−9^, 1 × 10^−8^, 5 × 10^−8^, and 1 × 10^−7^ M) (Figure 18b). The increase in C_RT0_ enhances the capturing capability of the T-line, resulting in a higher maximum value of the S_T_ curve, which generates a stronger signal. However, changes in C_RT0_ have no significant impact on the detection limits and working ranges calculated using the metrics in this study (Figure 18c).

The effect of C-line capture probe initial concentration, C_RC0_, on competitive LFIA performance is similar to that in the sandwich LFIA and will not be further elaborated here.

#### 4.3.5. The Influence of Reaction Constants on Competitive LFIA Performance

**Figure 19:**
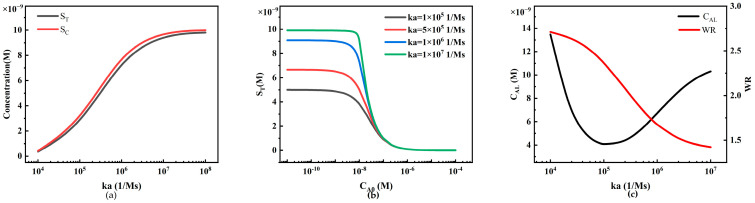
The relationship between association rate constant *ka* and LFIA detection performance: (**a**) Variations in S_T_ and S_C_ under different association rate constants *ka*; (**b**) inverse S-shaped standard curves under different association rate constants *ka*; (**c**) trends of LFIA detection limits and working ranges under different association rate constants *ka*.

**Figure 20:**
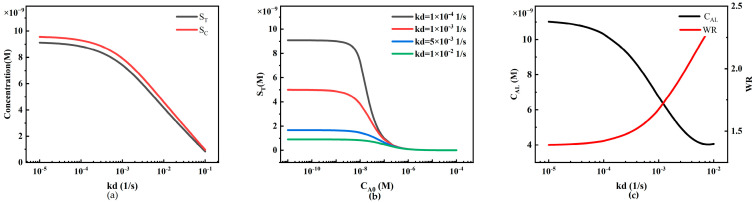
The relationship between dissociation rate constant *kd* and LFIA detection performance: (**a**) variations in S_T_ and S_C_ under different dissociation rate constants *kd*; (**b**) inverse S-shaped standard curves under different dissociation rate constants *kd*; (**c**) trends of LFIA detection limits and working ranges under different dissociation rate constants *kd*.

The effects of reaction constants *ka* and *kd* on competitive LFIA performance are depicted in Figure 19 and Figure 20. From Figure 19a and Figure 20a, we observe the variations in S_T_ and S_C_ at different values of *ka* and *kd* while holding the analyte concentration constant. Larger values of *ka* and lower values of *kd* enhance the efficiency of the reaction, resulting in higher S_T_ and S_C_ on both the T-line and C-line, which gradually saturate.

Figure 19b and Figure 20b depict the inverse S-curves under different binding rate constants (*ka* = 1 × 10^5^, 5 × 10^5^, 1 × 10^6^, 1 × 10^7^ M^−1^ s^−1^) and different dissociation constants (*kd* = 1 × 10^−4^, 1 × 10^−3^, 5 × 10^−3^, 1 × 10^−2^ s^−1^). Increasing *ka* and decreasing *kd* significantly narrow the working range (WR) (Figure 19c and Figure 20c). It also increases the system’s detection limit, which is detrimental to detection. These observations indicate that contrary to sandwich LFIA, increasing the binding rate *ka* and decreasing the dissociation rate *kd* can enhance the intensity of detection signals on both the T-line and C-line, but they have negative implications for the system’s detection limit and working range.

## 5. Conclusions

This paper uses the Richards equation to solve the capillary flow velocity model and couples the solved velocity field with the physical field of mass transfer in porous media to establish a three-dimensional LFIA model, investigating the reaction mechanism of LFIA under non-uniform flow conditions. The influence of pore size and porosity on flow velocity is explored, and the performance of LFIA under different average flow velocities is compared. Additionally, the transport and diffusion of various reaction components in sandwich LFIA and competitive LFIA are studied. Furthermore, the effects of analyte concentration, report particle concentration, capture probe concentration, and reaction constants on LFIA performance are analyzed. The conclusions are as follows:

(1) The sample flow velocity decreases exponentially with the distance from the sample front to the origin. Increasing the pore size and porosity of the membrane both increase the capillary flow velocity, thus reducing the sensitivity of LFIA.
(2) In sandwich LFIA, appropriately increasing the report particle concentration C_P0_, increasing the initial concentration of T-line capture probe C_RT0_, increasing the binding rate *ka*, and decreasing the dissociation rate *kd* are all beneficial for reducing the detection limit and broadening the working range of LFIA. The initial concentration of C-line capture probe C_RC0_ has little effect on LFIA performance but lowers the T/C ratio.
(3) For competitive LFIA, increasing the report particle concentration C_P0_, increasing the binding rate *ka*, and decreasing the dissociation rate *kd* may adversely affect the detection limit and working range of LFIA. Under the indicators of this paper, the effect of T-line C_RT0_ on LFIA performance is insensitive.

The three-dimensional simulation model proposed in this paper has multiple applications in LFIA. Firstly, it allows for a comprehensive understanding of LFIA’s flow characteristics and reaction mechanisms, thereby enabling the prediction of LFIA’s detection limit and working range, ultimately enhancing detection sensitivity and accuracy. Secondly, the model can guide the design and optimization of LFIA products, including optimizing fluid channel design and reagent injection methods to improve equipment performance and stability. Additionally, the model can be used to validate the effectiveness and feasibility of new LFIA technologies or improvement methods, facilitating the application and promotion of new technologies. Lastly, the model can provide recommendations for product improvement and optimization, such as adjusting reagent formulations and optimizing operational procedures to enhance product performance and competitiveness. In summary, the LFIA three-dimensional simulation model serves as a valuable tool and support for the research, development, and application of LFIA technology, with the potential to play a significant role in medical diagnosis, food safety, environmental monitoring, and other fields.

## Figures and Tables

**Table 1:** Main model parameters.

Parameters	Values	Descriptions
M_l	2 [cm]	NC membrane length
M_th	125 [m]	NC membrane thickness
L_th	1 [mm]	Line thickness
Poro	0.7	Porosity of NC membrane
d_p	0.45 [μm]	Pore diameter of NC membrane
κ	1.715 × 10^−14^ m^2^	Membrane permeability of NC membrane
Poro2	0.8	Porosity of other porous materials
d_p2	1 [μm]	Pore diameter of other porous materials
κ2	2.8444 × 10^−13^ m^2^	Membrane permeability of other porous materials
Hw	2 [mm]	Water head
p0	−4.06 × 10^−6^ N/m^2^	Richards equation initial phase pressure
C0	10^−8^ [M]	Concentration of substances

## Data Availability

The data that support the findings of this study are available within the article.
